# Characteristics of the 24-hour ambulatory blood pressure monitoring in a COVID-19 survivor

**DOI:** 10.2217/fca-2020-0235

**Published:** 2021-04-20

**Authors:** Daanyaal Wasim, Bjørn Alme, Stina Jordal, Tomas Mikal Lind Eagan, Marijana Tadic, Giuseppe Mancia, Anne Berit Guttormsen, Sahrai Saeed

**Affiliations:** ^1^Department of Heart Disease, Haukeland University Hospital, Bergen, Norway; ^2^Department of Anaesthesiology & Intensive Care Medicine, Haukeland University Hospital, Bergen, Norway; ^3^Department of Medicine, Haukeland University Hospital, Bergen, Norway; ^4^Department of Thoracic Medicine, Haukeland University Hospital, Bergen, Norway; ^5^Department of Cardiology, University Hospital “Dr. Dragisa Misovic - Dedinje”, Heroja Milana Tepica 1, Belgrade 11000, Serbia; ^6^University of Milano-Bicocca, Milano & Policlinico di Monza, Monza, Italy

**Keywords:** ambulatory blood pressure monitoring, case report, COVID-19, echocardiography, hypertension, prone positioning, systolic pulmonary artery pressure

## Abstract

COVID-19 infection primarily causes severe pneumonia complicated by acute respiratory distress syndrome and multiorgan failure requiring a ventilator support. We present a case of a 55-year-old male, admitted with COVID-19. He was obese but had no other medical conditions. His blood pressure was measured by his general physician on several occasions in the past, all values being normal (<140/90 mmHg). He developed multiorgan failure, requiring vasopressor and ventilator support for 17 days. A prone positioning improved the arterial oxygenation, and reduced the need for supplemental oxygen. After recovery, he showed persistently elevated blood pressure and sinus tachycardia both in clinic and out-of-clinic. The activation of the renin–angiotensin–aldosterone and sympathetic systems, volume-overload, hyperreninemia and cytokine storm might have contributed to the exaggerated cardiovascular response.

SARS coronavirus 2 (SARS-CoV-2) was discovered in the city of Wuhan, China in December 2019. The coronavirus disease 2019 (COVID-19), caused by SARS-CoV-2, rapidly spread to the entire world and in March 2020 the WHO officially declared this as a pandemic. Shortly after its outbreak, reports suggested that male sex and advanced age increased the risk of severe infection and mortality [1]. Furthermore, hypertension (although not adjusted for age and other unmeasured confounders), diabetes, obesity and cardiovascular disease were reported as the most common comorbid conditions among patients with COVID-19 infection with significant increased mortality [1–3]. COVID-19 infection primarily affects the lungs and cause severe pneumonia complicated by acute respiratory distress syndrome (ARDS), respiratory failure and shock requiring intensive care with ventilator support, as well as vasopressors and renal replacement therapy [4]. Neither angiotensin-converting enzyme inhibitors nor angiotensin receptor blockers are associated with increased risk of organ failure in COVID-19 disease [5]. Hence, patients with COVID-19 and elevated blood pressure (BP) are treated according to the current international guidelines [6]. However, how the fulminant course of this life-threatening disease and treatment in intensive care unit (ICU) affect the BP response and hypertension status in COVID-19 survivors is less known.

## Case presentation

Here, we present our experience from a 55-year-old male admitted to the emergency department after 7 days suffering from fever, chills, dry cough and abrupt worsening of shortness of breath. He was obese (BMI 42 kg/m^2^) but had otherwise no previous medical history of cardiovascular disease. On several occasions over a period of 15 years in the past, he had his BP measured by his general physician, all measurements being within normal range (<140/90 mmHg).

At admission, he had fever (38.5°C), tachycardia (heart rate of 114 bpm) and was cyanotic with a respiratory rate of 40–60/min and oxygen saturation of 50% while receiving 10 L oxygen on face mask (pO_2_/FiO_2_-index 8–9). His BP was 119/78 mmHg. The results of some essential blood tests are presented in Table 1. He was diagnosed with COVID-19 following a positive PCR for SARS-CoV-2 infection. His condition rapidly deteriorated as he developed ARDS and was immediately transferred to the ICU, intubated and put on ventilator. A chest x-ray showed severe bilateral pneumonia with patchy infiltration of both lungs, consistent with ARDS. A transthoracic echocardiogram, though quality was poor due to obesity and high-pressure ventilation, showed no sign of regional wall motion abnormalities. Left ventricular (LV) end-diastolic dimension was 5.1 cm and systolic function was normal. Tricuspid annular plane systolic excursion was 1.7 cm (normal ≥1.7 cm) and an estimated systolic pulmonary artery pressure of 50 mmHg.

**Table 1.** Laboratory tests during hospitalization.

Laboratory tests	Values^†^
Hemoglobin (g/dl)	9.3–12.3
Leucocytes (10**9)	23.4–5.0
Procalcitonin (mik/l)	2.98–0.11
C-reactive protein (mg/l)	216–5
Creatinine (mmol/l)	531–109
eGFR (ml/min/1.73 m^2^)	10–65
Troponin T (ng/l)	657–62
pro-BNP (ng/l)	>35000–150
Sodium (mmol/l)	147–142
Chloride (mmol/l)	112–102
Potassium (mmol/l)	3.9–4.4
D-Dimer (mg/l)	>4.0–0.6
International normalized ratio (INR)	1.3
Fibrinogen (g/l)	10.0–4.6
Total cholesterol (mmol/l)	4.6
LDL (mmol/l)	2.3
HDL (mmol/l)	0.9
Triglycerides (mmol/l)	3.2

† In more than one value, the first value denotes the highest or lowest abnormal value during hospitalization and the second on discharge.

eGFR: Estimated glomerular filtration rate.

**Figure 1.**
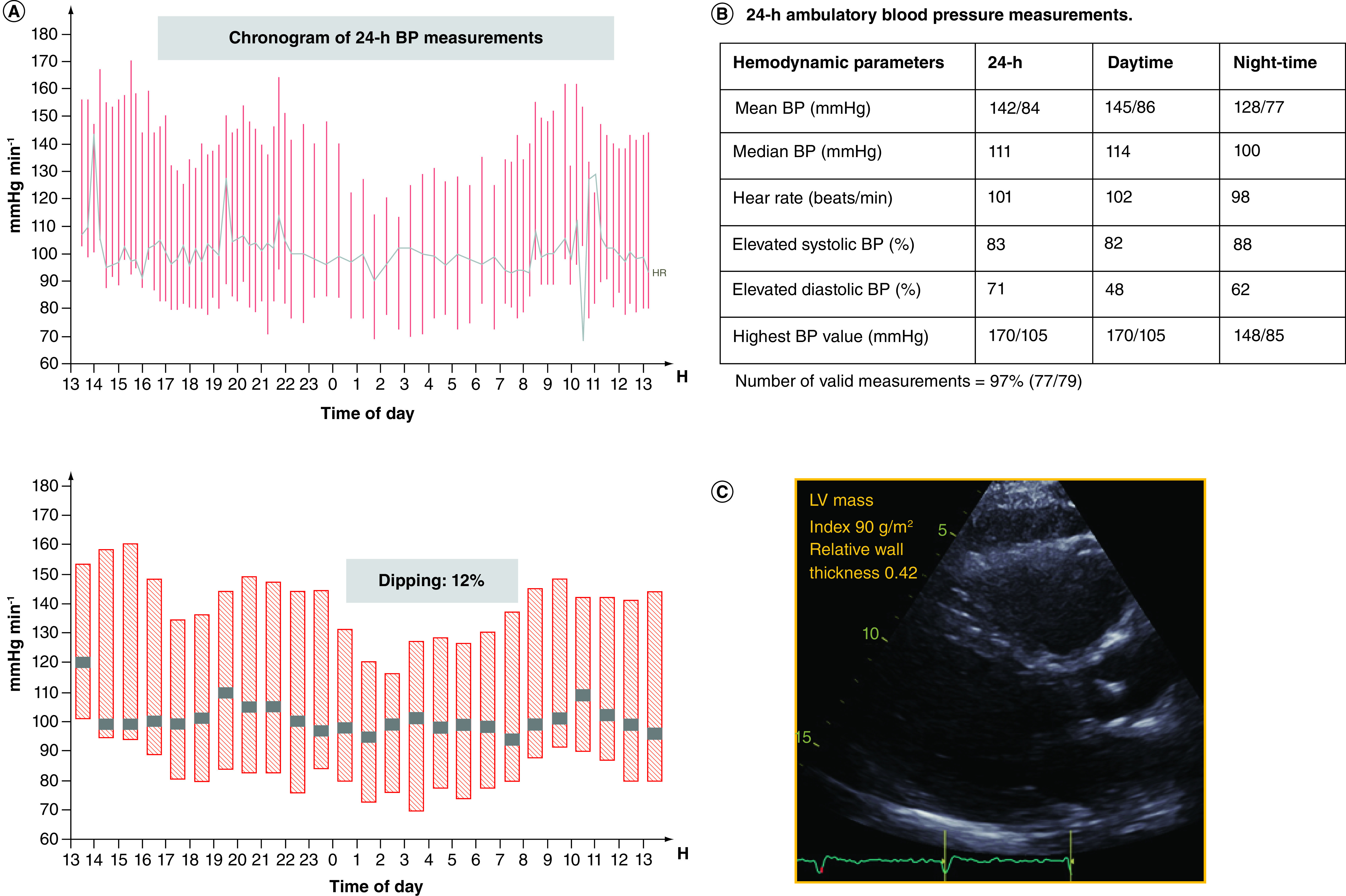
24-hour blood pressure measurements and echocardiographic image of the patient. **(A)** Chronogram of 24-hour BP measurements; **(B)** average 24-hour, daytime and night-time BP and heart rate; **(C)** transthoracic echocardiography, parasternal long-axis view showing normal left ventricular (LV) mass and relative wall thickness, and hence normal LV geometry. LV relative wall thickness is calculated as: (2 × posterior wall thickness)/(LV internal diameter at end-diastole). LV relative wall thickness is normal if ≤0.42 and increased if >0.42. Normal LV mass is ≤115 g/m^2^ in men and ≤95 g/m^2^ in women. BP: Blood pressure.

Based upon an inter-disciplinary discussion, he was treated with azithromycin and a third generation cephalosporin, hydroxychloroquine and IL-1 receptor antagonist. His condition deteriorated further and he developed severe multiorgan failure, including coagulopathy, heart failure and renal failure in addition to severe hypoxemia. He received a vasopressor support with norepinephrine for 15 days, vasopressine for 7 days and epinephrine for 2 days. Hemofiltration was also required. Due to severe and persistent hypoxemia, he was put in prone positioning intermittently for 6 days, after which a gradual improvement of his arterial oxygenation occurred and the need for supplemental oxygen was substantially reduced. He was treated on ventilator in pressure controlled and pressure support mode for 17 days and required positive airway pressure for an additional 8 days. A tracheostomy was performed on day-14. After 26 days in the ICU, he was transferred to the intermediate care ward and stayed there for another 2 weeks. His kidney function gradually recovered without need of further renal replacement therapy. He showed no sign of cognitive decline upon discharge. After a total of 40 days in hospital, he was transferred to a rehabilitation center, where he exhibited persistently elevated BP; on average systolic 160 and diastolic 90–110 mmHg and a sustained tachycardia with a resting heart rate of approximately 100 bpm. He was put on a beta-blocker and referred to an ambulatory 24-hour BP monitoring. This showed a mean 24-hour BP of 142/84 mmHg, daytime 145/86 mmHg and night-time 128/77 mmHg. 83% of systolic and 71% diastolic BP was elevated (≥130/80 mmHg) during 24-hour BP monitoring. He had sustained sinus tachycardia with a mean heart rate of 101 bpm over 24-hours. Although there was a 12% (normal) night-time BP dipping, the BP decline, however, occurred at late night and he had nocturnal hypertension with an exaggerated morning BP surge (Figure 1A–B). Due to elevated ambulatory BP measurements, he was put on ramipril 2.5 mg.

A follow-up echocardiogram showed normal LV geometry (LV mass index 90 g/m^2^ and relative wall thickness 0.42) (Figure 1C), complete normalization of systolic LV function (Simpson biplane ejection fraction of 65%) and regression of pulmonary hypertension. Left atrium anterior-posterior diameter was 3.3 cm, and LV end-diastolic diameter was 5.2 and end-systolic diameter 3.4 cm. Right ventricular size and function was normal: basal diameter 3.5 cm and tricuspid annular plane systolic excursion 2.7 cm. There was no sign of tricuspid regurgitation. Transmitral E wave velocity was 0.7 m/s, deceleration time 220 ms and A wave velocity 0.6 m/s. Septal and lateral annular e′ velocities were 7 cm/s and 10 cm/s, respectively. Filling pressure was normal (E/e′ ratio of 9). Right ventricular S′ was 15 cm/s and septal and lateral annular S′ 8 and 9 cm/s, respectively.

At a 4-months follow-up at his general physician, the BP was 157/107 mmHg. The dose of ramipril was increased to 5 mg.

## 6-month follow-up

At the 6-month follow-up, the patient was feeling well, did not have any breathing difficulty at rest and reported having resumed exercise and work. He had achieved BP control with clinic BP of 124/78 mmHg. A lung function test showed marked improvement of pulmonary function compared with the 3-months follow-up: Forced vital capacity of 4.3 l (80%), forced expiratory volume in 1 s (FEV_1_) 3.57 l (81%) and diffusion capacity (D_L_CO) 7.1 mmol/kPa.min (65%). A chest x-ray revealed significant regression of the infiltrations in the both lungs compared with chest x-ray taken at discharge. Renal function tests were normal: creatinine 111 μmol/l (60–105), estimated glomerular filtration rate 64 ml/min/1.73 m^2^, sodium 140 mmol/l (137–145) and potassium 4.1 mmol/l (3.5–5.0). A further 1-year follow-up is scheduled.

## Discussion

This case report illustrates two important findings: after survival from a severe life-threatening COVID-19 infection complicated by ARDS and multiple organ failure, our patient became sustained hypertensive throughout the convalescent period; prone positioning contributed to significant improvement of his arterial oxygenation, and the need for supplemental oxygen was substantially reduced due to less mismatch between ventilation and circulation [7-8].

After recovery, COVID-19 leads to worsening of hypertension, poor diabetic control and renal damage [2]. Hypertension and obesity are together with male sex common comorbidities and risk factors for unfavorable outcome in patients with COVID-19. Our patient had obesity and probably an underlying masked hypertension. However, the echocardiogram showed normal LV wall thicknesses and chamber dimensions. This excluded the possibility of previous significant hypertension and resultant target organ damage. Furthermore, after recovery from a life-threating COVID-19 infection complicated by severe ARDS and multiple organ failure, he developed sustained hypertension. His BP was persistently elevated both in clinic and out-of-clinic. We speculated the activation of neuro-endocrine hormone pathways, such as renin-aldosterone system and sympathetic system at periphery level in a high-risk patient of metabolic syndrome may have changed his hypertension status from masked to sustained hypertension. Furthermore, inflammation-induced systemic cytokine storm, immunological response, prolonged period of mechanical ventilation and associated sedation, volume-overload, use of inotropic agents, increased adrenergic tone, fever, hypoxia, inflammation, ischemia, vasculitis may also have contributed to an exaggerated cardiovascular response and worsening of hypertension. Although evidence from prospective studies are still not available, preliminary results based upon case series have recently indicated that patients with COVID-19 tend to develop hyperreninemia combined with hypernatremia and hyperchloremia in intensive care [9]. Notably, our patient also developed hypernatremia and hyperchloremia and acute kidney injury during intensive care. However, renin was not measured.

The hypertensive status prior to admission was based upon office BP and an ambulatory BP monitoring was not performed. Hence, it remains uncertain whether he had a truly masked hypertension (undetected established hypertension). However, after the COVID-19 infection and discharge from hospital, he showed worsening of hypertension with a sustained elevated BP. This observation is in line with current attention to the possibility that COVID-19 may lead to permanent or semipermanent post-COVID sequelae [2]. A 24-hour ambulatory BP monitoring should be performed to evaluate patient’s true hypertensive state in previously apparently normotensive patients and BP control in patients with known hypertension. A close follow-up with optimal antihypertensive treatment is crucial to avoid long-term cardiovascular complications in these patients.

The prognostic value of newly detected persistently elevated BP following hospitalization for COVID-19 infection should be explored in prospective studies in the future. Finally, the normalization of systolic pulmonary artery pressure measured by echocardiography was also an interesting finding. Acute respiratory failure/ARDS, inflammation, ischemia and vasculitis may be reasons for pulmonary hypertension in COVID-19 patients. Although right ventricular dysfunction and dilatation *per se* are markers of poor outcome [10,11], studies assessing the prognostic value of systolic pulmonary artery pressure regression are warranted.

## Conclusion

COVID-19 patients may undergo several hemodynamic changes during hospitalization affecting their BP level. In case of persistently elevated/fluctuating BP values in the clinic, an ambulatory BP monitoring should be performed to evaluate hypertension state, assess BP control and detect other risk features of 24-hour BP, such as nondipping pattern, nocturnal hypertension and morning surge, as well as assess heart rate response. The optimization of antihypertensive treatment and close follow-up at hypertension clinic is essential to achieve BP control and avoid hypertension-induced target organ damage in these vulnerable patients.

**Summary points**

- Acute cardiovascular complications in COVID-19 infection are common and include acute cardiac injury/COVID cardiomyopathy, thromboembolic complications, stroke and arrhythmias.
- Post-COVID-sequelae such as sustained elevated blood pressure, sinus tachycardia at rest, poor diabetic control and persistent renal damage may also occur.
- In intubated patients with severe acute respiratory distress syndrome, prone positioning contributes to significant improvement of arterial oxygenation and reduces the need for supplemental oxygen.
- In case of persistently elevated blood pressure and higher resting heart rate after recovery from COVID-19, an ambulatory blood pressure monitoring should be performed to evaluate patient’s true hypertensive state, assess blood pressure control and detect nondipping blood pressure pattern and nocturnal hypertension.

## References

[1] Guzik TJ, Mohiddin SA, Dimarco A COVID-19 and the cardiovascular system: implications for risk assessment, diagnosis, and treatment options. Cardiovasc. Res. 116, 1666–1687 (2020). 32352535 10.1093/cvr/cvaa106 PMC7197627

[2] Saeed S, Tadic M, Larsen TH, Grassi G, Mancia G. Coronavirus disease 2019 and cardiovascular complications: focused clinical review. J.Hypertens. (2021) (Epub ahead of print). 10.1097/HJH.0000000000002819 PMC9904438 33687179

[3] The European Society for Cardiology. ESC guidance for the diagnosis and management of CV disease during the COVID-19 pandemic (2020). https://www.escardio.org/Education/COVID-19-and-Cardiology/ESC-COVID-19-Guidance

[4] Fried JA, Ramasubbu K, Bhatt R The variety of cardiovascular presentations of COVID-19. Circulation 141, 1930–1936 (2020). 32243205 10.1161/CIRCULATIONAHA.120.047164 PMC7314498

[5] Hippisley-Cox J, Young D, Coupland C Risk of severe COVID-19 disease with ACE inhibitors and angiotensin receptor blockers: cohort study including 8.3 million people. Heart 106, 1503–1511 (2020). 32737124 10.1136/heartjnl-2020-317393 PMC7509391

[6] Williams B, Mancia G, Spiering W Task Force for the Management of Arterial Hypertension of the European Society of Hypertension; Task Force for the Management of Arterial Hypertension of the European Society of Cardiology. 2018 ESH/ESC guidelines for the management of arterial hypertension. J. Hypertens. 36, 1953–2041 (2018). 30234752 10.1097/HJH.0000000000001940

[7] Cohen D, Wasserstrum Y, Segev A Beneficial effect of awake prone position in hypoxaemic patients with COVID-19: case reports and literature review. Intern. Med. J. 50, 997–1000 (2020). 32697030 10.1111/imj.14926 PMC7404489

[8] Jiang LG, LeBaron J, Bodnar D Conscious proning: an introduction of a proning protocol for nonintubated, awake, hypoxic emergency department COVID-19 patients. Acad. Emerg. Med. 27, 566–569 (2020). 32462708 10.1111/acem.14035 PMC7283629

[9] Hulström M, von Seth M, Frithiof R. Hyperreninemia and low total body water may contribute to acute kidney injury in COVID-19 patients in intensive care unit. J. Hypertens. 38, 1613–1614 (2020). 32472780 10.1097/HJH.0000000000002531 PMC7282406

[10] Argulian E, Sud K, Vogel B Right ventricular dilation in hospitalized patients with COVID-19 infection. JACC Cardiovasc. Imaging. 13, 2459–2461 (2020). 32426088 10.1016/j.jcmg.2020.05.010 PMC7228729

[11] Li Y, Li H, Zhu S Prognostic value of right ventricular longitudinal strain in patients with COVID-19. JACC Cardiovasc. Imaging. 13, 2287–2299 (2020). 32654963 10.1016/j.jcmg.2020.04.014 PMC7195441

